# Supplementary material for: Why do ambulance employees (not) seek organisational help for mental health support? A mixed-methods systematic review protocol of organisational support available and barriers/facilitators to uptake
Source: BMJ Open. 2022 Oct 10;12(10):e062775. doi: 10.1136/bmjopen-2022-062775 (PMC9557302; doi:10.1136/bmjopen-2022-062775)
Supplement: Supplementary data [file bmjopen-2022-062775supp001.pdf]

Appendix 1: Medline search strategy

A draft search strategy in Medline for the following mixed-methods systematic review:

Why do ambulance employees (not) seek organisational help for mental health support?: A mixed-methods systematic review protocol of organisational support available and barriers/facilitators to uptake

Keywords

Emergency Medical Services; Mental Health; Organisational culture; Paramedical personnel; Systematic review

Search strategy

(Title/Abstract) (Medline):

1. “pre hospital"
2. pre-hospital
3. prehospital
4. paramedic*
5. ambulance*
6. aeromedical
7. “Aviation medicine”
8. HEMS
9. helicopter ADJ5 emergenc*
10. EMT
11. "emergency medical" ADJ technician*
12. ECA
13. "emergency care" ADJ assistant*
14. "Emergency call" ADJ (handler* OR operator*)
15. "Emergency dispatch*"

16. #1 OR #2 OR #3 OR #4 OR #5 OR #6 OR #7 OR #8 OR #9 OR #10 OR #11 OR #12 OR #13 OR #14 OR #15

---

17. anxiety
18. depress*
19. ptsd
20. "post-traumatic stress"
21. "post traumatic stress"
22. burnout
23. "burn out"
24. "self-harm"
25. self harm
26. "self injur*"
27. self-injur*
28. "self mutilat*"
29. self-mutilat*
30. distress
31. "mental health"
32. "mental illness*"
33. well-being
34. wellbeing
35. stress* suicid*
36. "critical incident stress"
37. #17 OR #18 OR #19 OR #20 OR #21 OR #22 OR #23 OR #34 OR #25 OR #26 OR #27 OR #28 OR #29 OR #30 OR #31 OR #32 OR #33 OR #34OR #35 OR #36

---

38. "occupational health"
39. "occupational mental health"
40. "occupational support"
41. "psychological support"
42. "psychological help"

43. help-seeking
44. help ADJ3 seeking
45. signposting
46. "employee assistance"
47. "employee support"
48. resilience
49. organi?ation* ADJ3 (support OR assistance)
50. work* ADJ3 (support OR assistance)
51. manager* ADJ3 (support OR assistance)
52. "crisis intervention"
53. downtime
54. surveil*
55. monitor*
56. #38 OR #39 OR #40 OR #41 OR #42 OR #43 OR #44 OR # 45 OR #46 OR #47 OR #48 OR #49 OR # 50 OR #51 OR #52 OR # 53 OR # 54 OR #55

---

57. #16 AND #37 AND #56
