# Supplementary material for: Why do ambulance employees (not) seek organisational help for mental health support? A mixed-methods systematic review protocol of organisational support available and barriers/facilitators to uptake
Source: BMJ Open. 2022 Oct 10;12(10):e062775. doi: 10.1136/bmjopen-2022-062775 (PMC9557302; doi:10.1136/bmjopen-2022-062775)
Supplement: Author's manuscript [file bmjopen-2022-062775.draft_revisions.pdf]

BMJ Open is committed to open peer review. As part of this commitment we make the peer review history of every article we publish publicly available.

When an article is published we post the peer reviewers' comments and the authors' responses online. We also post the versions of the paper that were used during peer review. These are the versions that the peer review comments apply to.

The versions of the paper that follow are the versions that were submitted during the peer review process. They are not the versions of record or the final published versions. They should not be cited or distributed as the published version of this manuscript.

BMJ Open is an open access journal and the full, final, typeset and author-corrected version of record of the manuscript is available on our site with no access controls, subscription charges or pay-per-view fees (http://bmjopen.bmj.com).

If you have any questions on BMJ Open's open peer review process please email info.bmjopen@bmj.com

BMJ Open

Why do ambulance employees (not) seek organisational help for mental health support? A mixed-methods systematic review protocol

Journal:	*BMJ Open*
Manuscript ID	bmjopen-2022-062775
Article Type:	Protocol
Date Submitted by the Author:	17-Mar-2022
Complete List of Authors:	Johnston, Sasha; South Western Ambulance Service NHS Foundation Trust, Bristol Station; University of Oxford, Department of Experimental Psychology<br>Sanderson, Kristy; University of East Anglia Faculty of Medicine and Health Sciences, Health Sciences; University of East Anglia, School of Health Sciences<br>Bowes, Lucy; University of Oxford, Department of Experimental Psychology<br>Wild, Jennifer; University of Oxford, Experimental Psychology; Oxford Health NIHR Biomedical Research Centre
Keywords:	MENTAL HEALTH, ACCIDENT & EMERGENCY MEDICINE, Organisation of health services < HEALTH SERVICES ADMINISTRATION & MANAGEMENT, Health & safety < HEALTH SERVICES ADMINISTRATION & MANAGEMENT

SCHOLARONE™
Manuscripts

Title:

Why do ambulance employees (not) seek organisational help for mental health support? A mixed-methods systematic review protocol

Author

Sasha Johnston[1,2]

Co-author's

Prof. Kristy Sanderson[4]

Prof. Lucy Bowes[2]

Dr. Jennifer Wild[2,3]

Institutional affiliations of authors:

[1]South Western Ambulance NHS Foundation Trust, Bristol, United Kingdom

[2]Department of Experimental Psychology, University of Oxford, Oxford, United Kingdom

[3]Oxford Health NIHR Biomedical Research Centre, Oxford, United Kingdom

[4] School of Health Sciences, University of East Anglia, Norwich, United Kingdom

Correspondence to Sasha Johnston;

Email: sasha.johnston@psy.ox.ac.uk

Mobile: 07825826250

Mailing address: Sasha Johnston, South Western Ambulance NHS Foundation Trust, North Education Centre (Block C), University of the West of England, Glenside Campus, Manor Road, Fishponds, Bristol, BS16 1DE

Ethics and dissemination Ethical approval is not required because only available published data will be analysed and this is a protocol for a systematic review. Findings will be disseminated through peer-reviewed publication and conference presentation.

Key words Emergency Medical Services; Mental Health; Organizational culture; Paramedical personnel; Systematic review

For peer review only

For peer review only - http://bmjopen.bmj.com/site/about/guidelines.xhtml

Abstract

Introduction

The COVID-19 pandemic is exacerbating a wide range of symptoms of poor mental health among emergency medical service (EMS) ambulance populations. Evidence suggests that utilising organisational support can improve employee outcomes and in turn, patient outcomes. Understanding why EMS staff do and do not utilize support services is therefore critical to improving uptake, ensuring equitable access, and potentially influencing workforce wellbeing, organisational sustainability, and patient care delivery. This systematic review aims to identify perceived barriers and facilitators to accessing and utilising organisational support.

Methods and Analysis

A search will be performed to identify studies that report barriers and facilitators to EMS employee support among all government/state commissioned EMS ambulance systems. Electronic databases, AMED, CINAHL, Cochrane Central Register of Controlled Trials, Cochrane Database of Systematic Reviews, EMBASE, EMCARE, HMIC, Medline and PsycINFO will be searched. All relevant English-language studies of adult employees of government/state commissioned EMS ambulance organisations published since December 2004 will be screened and relevant data extracted by two independent reviewers. A third reviewer will resolve any disagreements. The primary outcomes of this systematic review will include measures related to EMS ambulance staff perceived barriers or facilitators to accessing and utilising organisational assistance for mental health support. Study selection will follow Preferred Reporting Items for Systematic Reviews and Meta-Analyses (PRISMA) guidelines, and the methodological quality of included studies will be appraised by administering rating checklists. A narrative synthesis will be conducted to report qualitative and quantitative data and will include population characteristics, methodological approach and information about barriers and facilitators.

For peer review only - http://bmjopen.bmj.com/site/about/guidelines.xhtml

Ethics and Dissemination

Ethical approval is not required because only available published data will be analysed and this is a protocol for a systematic review. Findings will be disseminated through peer-reviewed publication and conference presentation.

Registration

PROSPERO registration number: CRD42022299650

Word count 2750

Article Summary

Strengths and limitations of this study

- This systematic review addresses a gap in the current evidence-base by providing an overview and critical appraisal of studies that report EMS employee perceptions of the barriers and facilitators to organisational mental health support, which may influence employee uptake of such support.
- By following the Preferred Reporting Items for Systematic Review and Meta-Analysis Protocols (PRISMA-P) and Synthesis without meta-analysis (SWiM) in systematic reviews reporting guidelines and by registering and publishing this protocol, we increase the transparency of our systematic review methods and findings.
- Restricting the study to English-language only publications may exclude relevant information written in other languages.
- There is potential for heterogeneous and low-quality reporting of barriers and facilitators in the studies identified for review.

For peer review only - http://bmjopen.bmj.com/site/about/guidelines.xhtml

Introduction

Emergency Medical Service (EMS) employees save lives. They respond to emergency and urgent care needs to reduce anxiety, pain, and suffering. EMS are called to work in a range of environments and with a range of patient populations, undertaking autonomous life and death decisions. They frequently work long, irregular hours, whilst contending with staff shortages and exposure to distressing and traumatic events. These factors can result in severe consequences for some staff; with an increased risk of post-traumatic stress, early retirement on medical grounds, accidental injury or death.[1] EMS employees are over four times more likely to experience mental ill health compared with the general workforce.[2,3] The COVID-19 pandemic is exacerbating risk of poor mental health. A recent survey of UK emergency responders identified that ambulance staff (77%) were the most likely to report their mental health has worsened since the pandemic began.[4] Suicide is a particular concern,[5] with Mars et al[6] identifying a 75% increased risk among male paramedics compared with the general population.

A number of risk factors contribute to EMS employee mental ill health, including those shared with the general population, such as genetics, loneliness, stressful life events and physical ill health.[7] A recent systematic review identified a higher prevalence of alcohol and drug misuse compared with the general population[3] and evidence suggests a high prevalence of adverse childhood experiences among EMS employees, such as abuse and neglect.[8] However, research by the mental health charity Mind[9] found that EMS employees were twice as likely as the general population to identify problems at work as the main cause of their mental ill health. Poor employee mental health can have a detrimental impact on EMS capability, with some areas reporting a 50% staff attrition rate, with poor staff mental health and organisational culture cited as primary contributing factors.[10,11] Evidence suggests that utilising organisational support when needed is related to improved employee and patient outcomes.[12] If support isn't available or employees are unable or won't access support, staff may feel isolated, unsupported and this can lead to poor mental health and an inability to thrive at work.[13] EMS employees report reluctance to disclose mental health problems at work, citing perceived stigma

For peer review only - http://bmjopen.bmj.com/site/about/guidelines.xhtml

associated with mental health and feeling unsupported by employers to address mental wellbeing. To help prevent workforce burnout, action is needed to better support EMS employee mental wellbeing. With the right support, staff experiencing mental ill health can successfully continue to work, the severity of symptoms can be reduced and suicide prevented.[14,15] In addition the frequency and length of sickness absence reduces; increasing workforce productivity, capability and safety.[16] Current EMS employee assistance programme uptake is improving, but it is vital that EMS organisations make improvements to ensure all employees can access support when needed.[17] This is vital not only because of the impact of poor mental health on individual employees, but also the critical impact of prehospital care on patient outcomes.[18] Therefore, understanding what EMS employees perceive to be barriers and facilitators to utilising support services is key to improving their uptake. This systematic review aims to improve our understanding of why some employees access organisational support and why others do not. This protocol aims to provide a transparent method of identifying barriers and facilitators whilst assessing the quality and risk of bias of the current available evidence.

Review aim

Our primary aim is to identify and review previously conducted studies which include reports of EMS ambulance employees' perceptions of the barriers or facilitators to the provision of organisational mental health support for their own psychological wellbeing.

Objectives

Our objective is to identify what element/s are perceived as effective and/or ineffective for the uptake and delivery of organisational support for EMS employee mental wellbeing. For the purposes of this review organisational support is defined as any program, pathway or signposting that is provided, funded or facilitated by the employing organisation in support of mental health. This review will seek to:

- Identify and report the range of the distinct types of supportive interventions available for EMS ambulance employee wellbeing

For peer review only - http://bmjopen.bmj.com/site/about/guidelines.xhtml

- Establish the proportions of participants that report barriers and/or facilitators and/or other key factors

- Identify attitudes, perceptions and experiences relating to any barriers, facilitators, and other key factors

Methods and analysis

We prepared this protocol following the Preferred Reporting Items for Systematic Review and Meta-Analysis Protocols (PRISMA-P) and Synthesis without meta-analysis (SWiM) in systematic reviews reporting guidelines.[19,20] The protocol was then registered with the International Prospective Register of Systematic Reviews (PROSPERO) on 2nd February 2022 (ref CRD42022299650).

Inclusion and exclusion criteria

Types of studies

All study types that examine factors relating to organisational mental health support for prehospital EMS ambulance organisation employees will be included. All types of relevant systematic review, quantitative, and mixed-methods studies will be included to assess barriers, facilitators, and any associated benefits and/or harms linked to reported interventions. Qualitative, cross-sectional and survey studies that report any barriers and/or facilitators relating to organisational employee mental health support, will also be examined.

We will only examine articles published after 1 December 2004, since this date coincides with a shift in focus on the wellbeing of first responders across the globe. This shift likely relates to the terrorist attack in New York on September 11th 2001. Legislative and guidance changes were introduced to ambulance organisations across the globe such as 'Agenda for change' (2004)[21] in the UK, in the United States of America the 'EMS Workforce for the 21st Century project'[22]

For peer review only - http://bmjopen.bmj.com/site/about/guidelines.xhtml

commenced in the fall of 2004 and in Australia the 'Emergencies Act (ACT)' (2004)[23] promoted responder welfare and described employer responsibility. We will exclude articles not written in English. Any study samples that consist of mixed emergency employees (ambulance/coastguard/fire/police), where results are combined, and samples include less than 50% ambulance staff will also be excluded from this systematic review.

Types of participants

All studies involving adults (18+) employed by government or state commissioned EMS organisations in clinical or non-clinical roles will be included. Employees will be eligible for inclusion if contracted to full or part-time roles or hold a bank contract that requires a minimum number of regular working hours. Employees could include paramedics, Emergency Medical Technicians, Emergency Care Assistants, EMS ambulance nurses and doctors, emergency medical number call centre and dispatch staff, operational managers, support and central function staff such as Human Resources and patient safety teams, as well as senior leadership. We will exclude paramedic students, EMS apprentices, non-government/state commissioned/private EMS ambulance employees and volunteers, including volunteer first responders, since any available supportive interventions may differ from those offered to employed staff.

Interventions

The review will include studies which report on EMS ambulance employees' perceived barriers or facilitators to seeking or accessing help from their organisation for mental health support. This may include individual level factors relating to the decision to engage in employee support, the acceptability of the support offered, perceptions and experiences of support, as well as organisational level factors such as, culture, and finally, policy level factors such as targeted campaigns and regulation of professional standards. Organisational factors examined in this review will include interventions reported to be offered for employee mental health and wellbeing. Any intervention, regardless of the mode of delivery (face-to-face, e-learning, virtual etc), is eligible for inclusion if the employer was involved in any element such as development,

For peer review only - http://bmjopen.bmj.com/site/about/guidelines.xhtml

design, delivery, funding, signposting. Studies that only examine social support (support outside of the employee context, such as non-organisational family and friend support) and organisational support in response to isolated specialist occurrences, such as natural disaster and terrorist events, will be excluded.

The main outcome will be the identification of EMS ambulance employees' perceived barriers or facilitators to accessing organisational support for their mental health (including formal peer-support networks, manager support and employee assistance programmes). This will Include elements of organisational factors identified by participants as being effective or ineffective for the provision and uptake of support. The presence of any factor that promotes the development, implementation, adoption, uptake of, or participation with, organisational employee mental health support will be considered a facilitator. Any factor that limits or restricts the development, implementation, adoption, uptake of or participation with organisational employee mental health support will be considered as a barrier. The same factor may be both a barrier and a facilitator.

Information sources

The following electronic databases will be searched: AMED, CINAHL, Cochrane Central Register of Controlled Trials and the Cochrane Database of Systematic Reviews via the Cochrane Library, EMBASE, EMCARE, HMIC, Medline, PsycINFO, Scopus and Web of Science. Searches will be tailored to each database using the Polyglot Search Translator[24] and conducted using keywords and relevant theasai such as MeSH and EMTREE. To ensure that we capture all the available and relevant research, grey literature will also be sought from the OpenGrey, MedNar and ProQuest databases and through the webpages of industry and charitable organisations active in supporting EMS ambulance employee mental health. A full list of webpages to be manually searched will be developed by the research team and will include sites such as the Global Ambulance Leadership Alliance (which covers the UK, US, Canada, and Australasia), The Ambulance Staff Charity (UK), the Royal Foundation, and the mental health charity, Mind.

For peer review only - http://bmjopen.bmj.com/site/about/guidelines.xhtml

Quantitative, qualitative, and mixed-method studies written in English since December 2004 will be included. This timeframe accounts for changes in policy that aimed to improve EMS ambulance employee working conditions, such as the UK's National Health Service 'Agenda for Change' which came into effect on 1 December 2004. The reference lists of all studies selected for critical appraisal will be hand searched for further material for inclusion. The searches will be re-run six weeks prior to the final analyses to identify and retrieve any other studies for inclusion.

For peer review only

Supplemental material

See Appendix 1: Example Medline OvidSP search strategy

Study Records

Data management

References identified from electronic and hand searches, including title and abstracts, will be imported into Mendeley citation manager software and any duplicates removed.

Selection process

Two reviewers will independently screen a subset (300) of titles and abstracts. Full-text screening will be based upon a PICoT concept:

- ***P****opulation*: Adults (18+) employed by government/state commissioned EMS ambulance services.
- *phenomena of* ***I****nterest*: Barriers and/or facilitators to organisational mental health support for ambulance staff.
- ***Co****ntext*: Government/state commissioned prehospital EMS ambulance organisations.
- ***T****ypes of studies study design*: All types of research studies.

For peer review only - http://bmjopen.bmj.com/site/about/guidelines.xhtml

Studies scoring 4/4 for all the above criteria will be included. Any reviewer uncertainty will be rated as 'unsure' and discussed by the independent reviewers with reference to the full text if required. If not resolved through reviewer discussion, disagreements will be settled through discussion with an independent third reviewer. The inter-rater reliability of consensus will be calculated.

Data extraction process

To identify papers for inclusion the full text of remaining studies will be retrieved and screened. Again, the inter-rater reliability will be calculated to ensure consistency and clarity. From this final selection, all potentially relevant data will be extracted and collated in an Excel spreadsheet including:

- Primary author
- Publication details
- Country of study
- Study methods
- Setting
- Sample characteristics (sample size, age range, EMS job role)
- Phenomenon of interest (self-reported barriers and/or facilitators)
- Intervention (where relevant)

Outcomes measured will include:

- Primary outcome measures (self-reported barriers and/or facilitators)
- Assessment tool names
- Reported statistics
- Reported significance levels
- Reported effect sizes
- Secondary outcome measures
- Relevant findings

For peer review only - http://bmjopen.bmj.com/site/about/guidelines.xhtml

For peer review only

To ensure sufficient detail capture to enable replication, any described intervention content will be extracted using Hoffman et al's[25] template for intervention description and replication (TIDieR) checklist. If data are missing or additional information is required, authors will be contacted. Search results will be reported in full and presented in a Preferred Reporting Items for Systematic Reviews and Meta-analyses (PRISMA) flow diagram.

Quality assessment

The quality, alongside the trustworthiness, relevance, and findings of each of the studies identified for final selection will be assessed by two independent reviewers using two rating checklists (Standard Quality Assessment Checklists) developed by Kmet, Cook and Lee.[26] One checklist is designed to assess the quality for quantitative studies (and will also be applied to the quantitative components of mixed-methods studies) and the other for qualitative studies (which will also be applied to the qualitative components of mixed-methods studies). Each checklist item will be rated on a quality scale from 0–2:

- criteria not met = 0
- criteria partially met = 1
- criteria fully met = 2

Any included grey literature will be assessed using Tyndall's[27] 'Authority, Accuracy, Coverage, Objectivity, Date, Significance' (AACODS) checklist. Reviewer discrepancies will be resolved through discussion and when necessary, consultation with the third reviewer. We plan to include all studies, regardless of the risk of bias, as it is anticipated that the availability of high-quality evidence will be limited. However, a sensitivity analysis will be conducted by removing any studies rated zero for quality, to determine the effect of excluding such studies in the results. Critical appraisal results will be displayed in a pre-determined 'risk of bias' table. The narrative will include a summary of the relative impact of missing data and of methodological flaws on the findings.

For peer review only - http://bmjopen.bmj.com/site/about/guidelines.xhtml

For peer review only

Data synthesis

Although mixed-methods systematic reviews are an emerging field of enquiry, this approach is useful for enhancing the credibility of findings. However, it is anticipated that data from the included studies will be heterogeneous since they are likely to include different approaches to design and use of different outcome measures. We will determine heterogeneity by summarising:

- Population characteristics (e.g., sample-size, age, type of mental health problem/disorder)
- Methodological approach (e.g., qualitative, survey, experiment)
- Assessment (the measures used to assess staff perceptions of organisational support, barriers or facilitators where relevant)
- Intervention characteristics (e.g., type of intervention, frequency, duration, uptake)

It is therefore unlikely that we will be able to undertake a meta-analysis. Instead, a narrative review and synthesis approach will be taken by conducting inductive thematic analysis, using NVivo software and data from the excel data extraction sheets in the following steps:

1. Key data and quotations will be transposed from data extraction sheets to NVivo for coding by two reviewers, who will agree a coding structure for coding of participant data. The third reviewer will arbitrate any conflict.

2. Using the agreed upon coding structure, the two reviewers will undertake thematic analysis of the coded data and will meet regularly to ensure the coding structure is appropriate and can be applied to the conclusions being drawn from the identified themes.

3. Factors impacting on participation with EMS organisational employee mental health support with a focus on information from studies relating to employee experiences and/or perceptions of barriers against, or facilitators to, accessing and utilising support,

For peer review only - http://bmjopen.bmj.com/site/about/guidelines.xhtml

will be synthesised in this systematic review (there will be no minimum number of studies).

4. We will use a narrative (descriptive) synthesis, following Campbell et al's[20] SWiM guideline.

5. The certainty of evidence will also be synthesised using #27 quality checklist.

6. We will seek to identify and describe relationships between the quantitative and qualitative data across all study types and present the results as a combined narrative, descriptive synthesis.

Amendments

If any protocol amendments are required, the date, description and rationale will be made available on the PROSPERO registration.

Patient and public involvement

To enhance the meaningfulness and robustness of findings, an EMS staff reference group and an EMS specific patient involvement group in the UK reviewed and supported the development and design of this protocol. These groups will review and provide an employee and public perspective on the interpretation of the findings and will support dissemination.

Ethics and dissemination

Ethical approval is not required because only available published data will be analysed and this is a protocol for a systematic review. Findings will be disseminated through publication in a relevant peer reviewed journal. The findings will also be communicated at research conferences, symposia, congresses and via social media to ensure dissemination to a wide range of interested parties.

For peer review only - http://bmjopen.bmj.com/site/about/guidelines.xhtml

Discussion

EMS employee mental wellbeing can influence the care given to patients. A number of initiatives are provided to support EMS employee mental health, although evidence suggests that some staff don't seek help or feel unable to disclose their mental health status when needed. With this in mind, a strength of this systematic review will be the presentation of barriers and facilitators specific to the uptake of employee mental health support in the EMS context identified through robust, replicable methods and critical appraisal of the available literature. Limitations will be addressed through transparent reporting and appraisal of study quality the involvement of EMS staff in the development of the inclusion and exclusion criteria, and by grading of the quality of the studies included.

For peer review only

For peer review only - http://bmjopen.bmj.com/site/about/guidelines.xhtml

Acknowledgements

We acknowledge the help and support of senior librarians Laura Coysh, Plymouth Discovery Library, Derriford Hospital, Plymouth and Karine Barker, Radcliffe Science Library, Bodleian libraries, University of Oxford. We are grateful for the funding provided by NHS England and NHS Horizons and for the support of South Western Ambulance NHS Foundation Trust.

Contributors

This study concept and design were conceived by authors SJ, JW and KS. SJ and JW drafted this manuscript with support from KS and LB who reviewed and edited the final version. All have approved the final submission.

Conflict of Interests statement

None declared

Funding statement

This work was supported by NHS England and NHS Horizons 'Project A' and by South Western Ambulance NHS Foundation Trust as part of SJ's DPhil in Experimental Psychology with the University of Oxford. JW is supported by MQ (CQRO1260), the Wellcome Trust (00070), and the Oxford Health NIHR Biomedical Research Centre.

For peer review only

References

1. Roy D, Weyman A, Nolan P. Self-preservation comes at a cost: Why British National Health Service paramedics might be choosing a healthier, but poorer, retirement. SAGE open Med [Internet]. 2020;8. Available from: https://www.ncbi.nlm.nih.gov/pmc/articles/PMC6977097/
2. Petrie K, Milligan-Saville J, Gayed A, Deady M, Phelps A, Dell L, et al. Prevalence of PTSD and common mental disorders amongst ambulance personnel: a systematic review and meta-analysis. Soc Psychiatry Psychiatr Epidemiol [Internet]. 2018 Sep 5 [cited 2021 Dec 16];53(9):897–909. Available from: http://www.ncbi.nlm.nih.gov/pubmed/29869691
3. Stevelink S, Pernet D, Dregan A, Davis K, Walker-Bone K, Fear NT, et al. The mental health of emergency services personnel in the UK Biobank: a comparison with the working population. Eur J Psychotraumatol [Internet]. 2020 Dec 31 [cited 2021 Dec 17];11(1). Available from: /pmc/articles/PMC7534319/?report=abstract
4. Mind. Behind the Mask: How the coronavirus pandemic impacted the mental health of emergency responders [Internet]. London; 2021 [cited 2021 Dec 13]. Available from: https://www.mind.org.uk/media/7246/blue-light-behind-the-mask-report-en-2021.pdf
5. James S. Prevention of Suicide in the Ambulance Service What We Know Health Education England 2 What We Know [Internet]. London; 2021 May [cited 2022 Jan 4]. Available from: https://aace.org.uk/wp-content/uploads/2021/05/CCS1120525712-003_Prevention_of_Suicide_in_the_Ambulance_Service_What_we_know_Web-Accessible.pdf
6. Mars B, Hird K, Bell F, James C, Gunnell D. Suicide among ambulance service staff: a review of coroner and employment records. Br Paramed J [Internet]. 2020 [cited 2021 Nov 6];4(4):10–5. Available from: https://doi.org/10.29045/14784726.2020.12.4.4.10
7. Wagner SL, White N, Regehr C, White M, Alden LE, Buys N, et al. Ambulance Personnel: Systematic Review of Mental Health Symptoms. Traumatology [Internet]. 2020 [cited 2021 Nov 28]; Available from: /record/2020-30911-001
8. Maunder RG, Halpern J, Schwartz B, Gurevich M. Symptoms and responses to critical

For peer review only - http://bmjopen.bmj.com/site/about/guidelines.xhtml

incidents in paramedics who have experienced childhood abuse and neglect. Emerg Med J. 2011;29(3):222–7.

9. Mind. Mental Health in the Emergency Services: Our 2019 Survey Results - Ambulance Service. London: Mind; 2019.

10. Willis E, Lawn S, Roberts L, Couzner L, Mohammadi L, Goble E. The impact of emergency call taking on the mental health and wellbeing of ambulance call-takers: A systematic thematic narrative of qualitative research. Australas J Paramed [Internet]. 2020 Jun 9 [cited 2021 Nov 29];17:1–11. Available from: https://doi.org/10.33151/ajp.17.801

11. South Western Ambulance NHS Foundation Trust. South Western Ambulance Service NHS Foundation Trust Annual Report and Accounts 1 April 2019 - 31 March 2020 [Internet]. Exeter; 2020 [cited 2021 Dec 29]. Available from: https://www.swast.nhs.uk/assets/1/swestamb_annual_report_with_accounts_2019-20.pdf

12. Gouweloos-Trines J, Tyler MP, Giummarra MJ, Kassam-Adams N, Landolt MA, Kleber RJ, et al. Perceived support at work after critical incidents and its relation to psychological distress: a survey among prehospital providers. Emerg Med J. 2017;34(12):816–22.

13. Stevenson D, Farmer P. Thriving at work [Internet]. London; 2017. Available from: https://assets.publishing.service.gov.uk/government/uploads/system/uploads/attachment_data/file/658145/thriving-at-work-stevenson-farmer-review.pdf

14. World Health Organization. Preventing Suicide: A global imperative [Internet]. 2014 [cited 2022 Jan 10]. Available from: https://apps.who.int/iris/bitstream/handle/10665/131056/9789241564779_eng.pdf;jsessionid=B4E631D830D2F3E40760A7E2416C01BF?sequence=1

15. Walumbwa FO, Muchiri MK, Misati E, Wu C, Meiliani M. Inspired to perform: A multilevel investigation of antecedents and consequences of thriving at work. J Organ Behav [Internet]. 2018 Mar 1 [cited 2021 Dec 17];39(3):249–61. Available from: http://doi.wiley.com/10.1002/job.2216

16. Hampson E, Jacob A. Mental health and employers; Refreshing the case for investment [Internet]. London; 2020. Available from:

For peer review only - http://bmjopen.bmj.com/site/about/guidelines.xhtml

https://www2.deloitte.com/content/dam/Deloitte/uk/Documents/consultancy/deloitte-uk-mental-health-and-employers.pdf

17. House of Commons. Workforce burnout and resilience in the NHS and social care Second Report of Session 2021-22 Report, together with formal minutes relating to the report [Internet]. London; 2021 May [cited 2022 Jan 2]. Available from: www.parliament.uk/hsccom

18. NHS Improvement. Operational productivity and performance in English NHS Ambulance Trusts: Unwarranted variations [Internet]. 2019. Available from: https://www.england.nhs.uk/wp-content/uploads/2019/09/Operational_productivity_and_performance_NHS_Ambulance_Trusts_final.pdf

19. Shamseer L, Moher D, Clarke M, Ghersi D, Liberati A, Petticrew M, et al. Preferred reporting items for systematic review and meta-analysis protocols (PRISMA-P) 2015: elaboration and explanation. BMJ [Internet]. 2015 Jan 2 [cited 2022 Jan 20];350:g7647. Available from: http://www.ncbi.nlm.nih.gov/pubmed/25555855

20. Campbell M, McKenzie JE, Sowden A, Katikireddi SV, Brennan SE, Ellis S, et al. Synthesis without meta-analysis (SWiM) in systematic reviews: reporting guideline. BMJ [Internet]. 2020 Jan 16 [cited 2022 Jan 28];368. Available from: https://www.bmj.com/content/368/bmj.l6890

21. NHS Employers. Agenda for Change: NHS TERMS AND CONDITIONS OF SERVICE HANDBOOK [Internet]. Leeds; 2004. Available from: https://www.nhsemployers.org/sites/default/files/2021-06/tcs-handbook-version-2004.pdf

22. National Highway Traffic Safety Administration. EMS Workforce for the 21st Century: A National Assessment [Internet]. Washington, D.C.; 2008. Available from: https://www.google.com/url?sa=t&rct=j&q=&esrc=s&source=web&cd=&ved=2ahUKEwjFo8G6lbT2AhWPa8AKHQ6sDZ0QFnoECAsQAQ&url=https%3A%2F%2Fwww.ems.gov%2Fpdf%2Fresearch%2FStudies-and-Reports%2FNational_Workforce_Assessment.pdf&usg=AOvVaw3bI-r62sS-46Z8qX7F-c7C

For peer review only - http://bmjopen.bmj.com/site/about/guidelines.xhtml

23. ACT Parliamentary Counsel. Emergencies Act 2004: A2004-28 [Internet]. Canberra; 2004. Available from: https://www.legislation.act.gov.au/DownloadFile/a/2004-28/current/PDF/2004-28.PDF

24. Clark J, Carter M, Honeyman D, Cleo G, Auld Y, Booth D, Condron P, Dalais C, Dern S, Linthwaite B, May N, Munn J, Ramsay L, Rickett K, Rutter C, Sanders S, Sondergeld P, Wallin M BE. The Polyglot Search Translator (PST): evaluation of a tool for improving searching in systematic reviews: a randomised cross-over trial. In: Abstracts of the 25th Cochrane Colloquium. Edinburgh: Cochrane Database of Systematic Reviews; 2018.

25. Hoffmann TC, Glasziou PP, Boutron I, Milne R, Perera R, Moher D, et al. Better reporting of interventions: template for intervention description and replication (TIDieR) checklist and guide. BMJ [Internet]. 2014 Mar 7 [cited 2021 Dec 28];348. Available from: https://www.bmj.com/content/348/bmj.g1687

26. Kmet LM, Cook LS, Lee RC. Standard Quality Assessment Criteria for Evaluating Primary Research Papers from a Variety of Fields [Internet]. Vol. 13, Alberta Heritage Foundation for Medical Research (AHFMR). Edmonton; 2004 Feb [cited 2021 Nov 20]. Available from: https://era.library.ualberta.ca/items/48b9b989-c221-4df6-9e35-af782082280e

27. Tyndall J. AACODS Checklist [Internet]. Adelaide; 2010 [cited 2022 Jan 3]. Available from: https://dspace.flinders.edu.au/xmlui/bitstream/handle/2328/3326/AACODS_Checklist.pdf?sequence=4&isAllowed=y

For peer review only

For peer review only - http://bmjopen.bmj.com/site/about/guidelines.xhtml

Appendix 1

Medline search strategy

Draft for the search strategy for our mixed-methods systematic review of why do ambulance employees (not) seek organisational help for mental health support?

Keywords

Systematic review; mental health; ambulance; paramedic; Emergency Medical Service; organisational support, thematic analysis

Search strategy

(Title/Abstract) (Medline):

1. “pre hospital"
2. pre-hospital
3. prehospital
4. paramedic*
5. ambulance*
6. aeromedical
7. “Aviation medicine”
8. HEMS
9. helicopter ADJ5 emergenc*
10. EMT
11. "emergency medical" ADJ technician*
12. ECA
13. "emergency care" ADJ assistant*
14. "Emergency call" ADJ (handler* OR operator*)
15. "Emergency dispatch*"
16. #1 OR #2 OR #3 OR #4 OR #5 OR #6 OR #7 OR #8 OR #9 OR #10 OR #11 OR #12 OR #13 OR #14 OR #15

---

17. anxiety

For peer review only - http://bmjopen.bmj.com/site/about/guidelines.xhtml

18. depress*
19. ptsd
20. "post-traumatic stress"
21. "post traumatic stress"
22. burnout
23. "burn out"
24. "self-harm"
25. self harm
26. "self injur*"
27. self-injur*
28. "self mutilat*"
29. self-mutilat*
30. distress
31. "mental health"
32. "mental illness*"
33. well-being
34. wellbeing
35. stress* suicid*
36. "critical incident stress"
37. #17 OR #18 OR #19 OR #20 OR #21 OR #22 OR #23 OR #34 OR #25 OR #26 OR #27 OR #28 OR #29 OR #30 OR #31 OR #32 OR #33 OR #34OR #35 OR #36

---

38. "occupational health"
39. "occupational mental health"
40. "occupational support"
41. "psychological support"
42. "psychological help"
43. help-seeking
44. help ADJ3 seeking
45. signposting

46. "employee assistance"
47. "employee support"
48. resilience
49. organi?ation* ADJ3 (support OR assistance)
50. work* ADJ3 (support OR assistance)
51. manager* ADJ3 (support OR assistance)
52. "crisis intervention"
53. downtime
54. surveil*
55. monitor*
56. #38 OR #39 OR #40 OR #41 OR #42 OR #43 OR #44 OR # 45 OR #46 OR #47 OR #48 OR #49 OR # 50 OR #51 OR #52 OR # 53 OR # 54 OR #55

---

57. #16 AND #37 AND #56

---

eview only

PRISMA-P (Preferred Reporting Items for Systematic review and Meta-Analysis Protocols) 2015 checklist: recommended items to address in a systematic review protocol*

Section and topic	Item No	Checklist item	Section
**ADMINISTRATIVE INFORMATION**			
**Title: Why do ambulance employees (not) seek organisational help for mental health support? A mixed-methods systematic review protocol**			
Identification	1a	Identify the report as a protocol of a systematic review	Title p.1
Update	1b	If the protocol is for an update of a previous systematic review, identify as such	n/a
Registration	2	If registered, provide the name of the registry (such as PROSPERO) and registration number	
Authors:			
Contact	3a	Provide name, institutional affiliation, e-mail address of all protocol authors; provide physical mailing address of corresponding author	Title page p.1
Contributions	3b	Describe contributions of protocol authors and identify the guarantor of the review	Contributor statement p.15
Amendments	4	If the protocol represents an amendment of a previously completed or published protocol, identify as such and list changes; otherwise, state plan for documenting important protocol amendments	n/a
Support:			
Sources	5a	Indicate sources of financial or other support for the review	Funding statement p.15
Sponsor	5b	Provide name for the review funder and/or sponsor	
Role of sponsor or funder	5c	Describe roles of funder(s), sponsor(s), and/or institution(s), if any, in developing the protocol	
**INTRODUCTION**			
Rationale	6	Describe the rationale for the review in the context of what is already known	Introduction pp.4-5
Objectives	7	Provide an explicit statement of the question(s) the review will address with reference to participants, interventions, comparators, and outcomes (PICO)	Aim and objectives pp.5-6 & Selection process p.9
**METHODS**			
Eligibility criteria	8	Specify the study characteristics (such as PICO, study design, setting, time frame) and report characteristics (such as years considered, language, publication status) to be used as criteria for eligibility for the review	Methods pp.6-9

For peer review only

Information sources	9	Describe all intended information sources (such as electronic databases, contact with study authors, trial registers or other grey literature sources) with planned dates of coverage	Methods pp.7-8
Search strategy	10	Present draft of search strategy to be used for at least one electronic database, including planned limits, such that it could be repeated	Supplementary appendix 1
Study records:			
Data management	11a	Describe the mechanism(s) that will be used to manage records and data throughout the review	Data management pp.9-10
Selection process	11b	State the process that will be used for selecting studies (such as two independent reviewers) through each phase of the review (that is, screening, eligibility and inclusion in meta-analysis)	Data management/ synthesis pp.9-13
Data collection process	11c	Describe planned method of extracting data from reports (such as piloting forms, done independently, in duplicate), any processes for obtaining and confirming data from investigators	Data management/ synthesis pp.9-13
Data items	12	List and define all variables for which data will be sought (such as PICO items, funding sources), any pre-planned data assumptions and simplifications	Data management/ synthesis pp.9-13
Outcomes and prioritization	13	List and define all outcomes for which data will be sought, including prioritization of main and additional outcomes, with rationale	Data synthesis pp.10-12
Risk of bias in individual studies	14	Describe anticipated methods for assessing risk of bias of individual studies, including whether this will be done at the outcome or study level, or both; state how this information will be used in data synthesis	Quality assessment & data synthesis pp.11-13
Data synthesis	15a	Describe criteria under which study data will be quantitatively synthesised	
	15b	If data are appropriate for quantitative synthesis, describe planned summary measures, methods of handling data and methods of combining data from studies, including any planned exploration of consistency (such as I^2, Kendall's τ)	Data synthesis pp.11-13
	15c	Describe any proposed additional analyses (such as sensitivity or subgroup analyses, meta-regression)	
	15d	If quantitative synthesis is not appropriate, describe the type of summary planned	
Meta-bias(es)	16	Specify any planned assessment of meta-bias(es) (such as publication bias across studies, selective reporting within studies)	Quality assessment p.11
Confidence in cumulative evidence	17	Describe how the strength of the body of evidence will be assessed (such as GRADE)	Quality assessment p.11

*** It is strongly recommended that this checklist be read in conjunction with the PRISMA-P Explanation and Elaboration (cite when available) for important clarification on the items. Amendments to a review protocol should be tracked and dated. The copyright for PRISMA-P (including checklist) is held by the PRISMA-P Group and is distributed under a Creative Commons Attribution Licence 4.0.**

From: Shamseer L, Moher D, Clarke M, Ghersi D, Liberati A, Petticrew M, Shekelle P, Stewart L, PRISMA-P Group. Preferred reporting items for systematic review and meta-analysis protocols (PRISMA-P) 2015: elaboration and explanation. BMJ. 2015 Jan 2;349(jan02 1):g7647.

For peer review only

BMJ Open

Why do ambulance employees (not) seek organisational help for mental health support? A mixed-methods systematic review protocol of organisational support available and barriers/facilitators to uptake

Journal:	*BMJ Open*
Manuscript ID	bmjopen-2022-062775.R1
Article Type:	Protocol
Date Submitted by the Author:	17-Aug-2022

Complete List of Authors:	Johnston, Sasha; South Western Ambulance Service NHS Foundation Trust, Bristol Station; University of Oxford, Department of Experimental Psychology<br>Sanderson, Kristy; University of East Anglia Faculty of Medicine and Health Sciences, Health Sciences; University of East Anglia, School of Health Sciences<br>Bowes, Lucy; University of Oxford, Department of Experimental Psychology<br>Wild, Jennifer; University of Oxford, Experimental Psychology; Oxford Health NIHR Biomedical Research Centre

**Primary Subject Heading**:	Mental health
Secondary Subject Heading:	Emergency medicine, Health services research, Qualitative research, Evidence based practice
Keywords:	MENTAL HEALTH, ACCIDENT & EMERGENCY MEDICINE, Organisation of health services < HEALTH SERVICES ADMINISTRATION & MANAGEMENT, Health & safety < HEALTH SERVICES ADMINISTRATION & MANAGEMENT, Human resource management < HEALTH SERVICES ADMINISTRATION & MANAGEMENT

SCHOLARONE™
Manuscripts

Title:

Why do ambulance employees (not) seek organisational help for mental health support? A mixed-methods systematic review protocol of organisational support available and barriers/facilitators to uptake

Author

Sasha Johnston[1,2]

Co-author's

Prof. Kristy Sanderson[4]

Prof. Lucy Bowes[2]

Dr. Jennifer Wild[2,3]

Institutional affiliations of authors:

[1]South Western Ambulance NHS Foundation Trust, Bristol, United Kingdom

[2]Department of Experimental Psychology, University of Oxford, Oxford, United Kingdom

[3]Oxford Health NIHR Biomedical Research Centre, Oxford, United Kingdom

[4] School of Health Sciences, University of East Anglia, Norwich, United Kingdom

Correspondence to Sasha Johnston;

Email: sasha.johnston@psy.ox.ac.uk

Mobile: 07825826250

Mailing address: Sasha Johnston, South Western Ambulance NHS Foundation Trust, North Education Centre (Block C), University of the West of England, Glenside Campus, Manor Road, Fishponds, Bristol, BS16 1DE

Ethics and dissemination Ethical approval is not required because only available published data will be analysed and this is a protocol for a systematic review. Findings will be disseminated through peer-reviewed publication and conference presentation.

For peer review only

For peer review only - http://bmjopen.bmj.com/site/about/guidelines.xhtml

Key words Emergency Medical Services; Mental Health; Organisational culture; Paramedical personnel; Systematic review

Abstract

Introduction

The COVID-19 pandemic is exacerbating a wide range of symptoms of poor mental health among emergency medical service (EMS) ambulance populations. Evidence suggests that utilising organisational support can improve employee outcomes and in turn, patient outcomes. Understanding why EMS staff do and do not utilise support services is therefore critical to improving uptake, ensuring equitable access, and potentially influencing workforce wellbeing, organisational sustainability, and patient care delivery. This systematic review aims to identify what support is available and any perceived barriers and facilitators to accessing and utilising organisational support.

Methods and Analysis

Searches performed between 18th-23rd February 2022 will be used to identify studies that report barriers and facilitators to EMS employee support among all government/state commissioned EMS ambulance systems. Electronic databases, AMED, CINAHL, Cochrane Central Register of Controlled Trials, Cochrane Database of Systematic Reviews, EMBASE, EMCARE, HMIC, Medline and PsycINFO will be searched. All relevant English-language studies of adult employees of government/state commissioned EMS ambulance organisations published since December 2004 will be screened and relevant data extracted by two independent reviewers. A third reviewer will resolve any disagreements.

The primary outcome is the identification of perceived barriers or facilitators to EMS staff utilising organisational support for mental health. The secondary outcome is the identification of supportive interventions offered through or by ambulance Trusts. Study selection will follow Preferred Reporting Items for Systematic Reviews and Meta-Analyses (PRISMA) guidelines, and

For peer review only - http://bmjopen.bmj.com/site/about/guidelines.xhtml

the methodological quality of included studies will be appraised by administering rating checklists. A narrative synthesis will be conducted to report qualitative and quantitative data and will include population characteristics, methodological approach and information about barriers and facilitators.

Ethics and Dissemination

Ethical approval is not required because only available published data will be analysed. Findings will be disseminated through peer-reviewed publication and conference presentation.

Registration

PROSPERO registration number: CRD42022299650

Word count 3014

Article Summary

Strengths and limitations of this study

- This systematic review addresses a gap in the current evidence-base by providing an overview and critical appraisal of studies that report EMS employee perceptions of the barriers and facilitators to organisational mental health support, which may influence employee uptake of such support.

- By following the Preferred Reporting Items for Systematic Review and Meta-Analysis Protocols (PRISMA-P) and Synthesis without meta-analysis (SWiM) in systematic reviews reporting guidelines and by registering and publishing this protocol, the transparency of systematic review methods and findings is improved.

- Restricting the study to English-language only publications may exclude relevant information written in other languages.

For peer review only - http://bmjopen.bmj.com/site/about/guidelines.xhtml

For peer review only

- There is potential for heterogeneous and low-quality reporting of barriers and facilitators in the studies identified for review.

For peer review only

For peer review only - http://bmjopen.bmj.com/site/about/guidelines.xhtml

Introduction

Emergency Medical Service (EMS) employees save lives. They respond to emergency and urgent care needs to reduce anxiety, pain, and suffering. EMS are called to work in a range of environments and with a range of patient populations, undertaking autonomous life and death decisions. They frequently work long, irregular hours, whilst contending with staff shortages and exposure to distressing and traumatic events. These factors can result in severe consequences for some staff; with an increased risk of post-traumatic stress, early retirement on medical grounds, accidental injury or death.[1] EMS employees are over four times more likely to experience mental ill health compared with the general workforce.[2,3] The COVID-19 pandemic is exacerbating risk of poor mental health. A recent survey of UK emergency responders identified that ambulance staff (77%) were the most likely to report their mental health has worsened since the pandemic began.[4] Suicide is a particular concern,[5] with Mars et al[6] identifying a 75% increased risk among male paramedics compared with the general population.

A number of risk factors contribute to EMS employee mental ill health, including those shared with the general population, such as genetics, loneliness, stressful life events and physical ill health.[7] A recent systematic review identified a higher prevalence of alcohol and drug misuse compared with the general population[3] and evidence suggests a high prevalence of adverse childhood experiences among EMS employees, such as abuse and neglect.[8] However, research by the mental health charity Mind[9] found that EMS employees were twice as likely as the general population to identify problems at work as the main cause of their mental ill health. Poor employee mental health can have a detrimental impact on EMS capability, with some areas reporting a 50% staff attrition rate, with poor staff mental health and organisational culture cited as primary contributing factors.[10,11] Evidence suggests that utilising organisational support when needed is related to improved employee and patient outcomes.[12] If support isn't available or employees are unable or won't access support, staff

For peer review only - http://bmjopen.bmj.com/site/about/guidelines.xhtml

may feel isolated, unsupported and this can lead to poor mental health and an inability to thrive at work.[13] EMS employees report reluctance to disclose mental health problems at work, citing perceived stigma associated with mental health and feeling unsupported by employers to address mental wellbeing. To help prevent workforce burnout, action is needed to better support EMS employee mental wellbeing. With the right support, staff experiencing mental ill health can successfully continue to work, the severity of symptoms can be reduced and suicide prevented.[14,15] In addition the frequency and length of sickness absence reduces; increasing workforce productivity, capability and safety.[16] Current EMS employee assistance programme uptake is improving, but it is vital that EMS organisations make improvements to ensure all employees can access support when needed.[17] This is vital not only because of the impact of poor mental health on individual employees, but also the critical impact of prehospital care on patient outcomes.[18] Therefore, understanding what EMS employees perceive to be barriers and facilitators to utilising support services is key to improving their uptake. This systematic review aims to improve our understanding of why some employees access organisational support and why others do not. This protocol aims to provide a transparent method of identifying current support provision, barriers and facilitators to utilising support, whilst assessing the quality and risk of bias of the current available evidence.

Review aim

Our primary aim is to identify and review previously conducted studies which include reports of EMS ambulance employees' perceptions of the barriers or facilitators to the provision of organisational mental health support for their own psychological wellbeing.

Objectives

The objective is to establish what support is available and identify any element/s perceived as effective and/or ineffective for the uptake of organisational support for EMS employee mental

For peer review only - http://bmjopen.bmj.com/site/about/guidelines.xhtml

wellbeing. For the purposes of this review organisational support is defined as any program, pathway or signposting that is provided, funded or facilitated by the employing organisation in support of mental health. This review will seek to:

- Identify and report the range of the distinct types of supportive interventions available for EMS ambulance employee wellbeing
- Establish the proportions of participants that report barriers and/or facilitators and/or other key factors
- Identify attitudes, perceptions and experiences relating to any barriers, facilitators, and other key factors

Methods and analysis

This protocol was prepared following the Preferred Reporting Items for Systematic Review and Meta-Analysis Protocols (PRISMA-P) and Synthesis without meta-analysis (SWiM) in systematic reviews reporting guidelines.[19,20] The protocol was then registered with the International Prospective Register of Systematic Reviews (PROSPERO) on 2nd February 2022 (ref CRD42022299650).

Inclusion and exclusion criteria

Types of studies

All study types that examine factors relating to organisational mental health support for prehospital EMS ambulance organisation employees will be included. Primary papers from relevant systematic reviews alongside quantitative, and mixed-methods studies will be included to establish what interventions are offered and to assess barriers, facilitators, and any associated benefits and/or harms linked to reported interventions. Qualitative, cross-sectional

For peer review only - http://bmjopen.bmj.com/site/about/guidelines.xhtml

and survey studies that report any barriers and/or facilitators relating to organisational employee mental health support, will also be examined.

Only articles published after 1 December 2004 will be examined, since this date coincides with a shift in focus on the wellbeing of first responders across the globe. This shift likely relates to the terrorist attack in New York on September 11th 2001. Legislative and guidance changes were introduced to ambulance organisations across the globe such as 'Agenda for change' (2004)[21] in the UK, in the United States of America the 'EMS Workforce for the 21st Century project'[22] commenced in the fall of 2004 and in Australia the 'Emergencies Act (ACT)' (2004)[23] promoted responder welfare and described employer responsibility. Articles not written in English will be excluded. Any study samples that consist of mixed emergency employees (ambulance/ coastguard/fire/police), where results are combined, and samples include less than 50% ambulance staff will also be excluded from this systematic review.

Types of participants

All studies involving adults (18+) employed by government or state commissioned EMS organisations in clinical or non-clinical roles will be included. Employees will be eligible for inclusion if contracted to full or part-time roles or hold a bank contract that requires a minimum number of regular working hours. Employees could include paramedics, Emergency Medical Technicians, Emergency Care Assistants, EMS ambulance nurses and doctors, emergency medical number call centre and dispatch staff, operational managers, support and central function staff such as Human Resources and patient safety teams, as well as senior leadership. Paramedic students, EMS apprentices, non-government/state commissioned/private EMS ambulance employees and volunteers, including volunteer first responders will be excluded, since any available supportive interventions may differ from those offered to employed staff.

Interventions

For peer review only - http://bmjopen.bmj.com/site/about/guidelines.xhtml

The review will include studies which report on EMS ambulance employees' perceived barriers or facilitators to seeking or accessing help from their organisation for mental health support. This may include individual level factors relating to the decision to engage in employee support, the acceptability of the support offered, perceptions and experiences of support, as well as organisational level factors such as, culture, and finally, policy level factors such as targeted campaigns and regulation of professional standards. Organisational factors examined in this review will include interventions reported to be offered for employee mental health and wellbeing. Any intervention, regardless of the mode of delivery (face-to-face, e-learning, virtual etc), is eligible for inclusion if the employer was involved in any element such as development, design, delivery, funding, signposting. Studies that only examine social support (support outside of the employee context, such as non-organisational family and friend support) and organisational support in response to isolated specialist occurrences, such as natural disaster and terrorist events, will be excluded.

The main outcome will be the identification of EMS ambulance employees' perceived barriers or facilitators to accessing organisational support for their mental health (including formal peer-support networks, manager support and employee assistance programmes). This will Include elements of organisational factors identified by participants as being effective or ineffective for the provision and uptake of support. The presence of any factor *that* promotes the development, implementation, adoption, uptake of, or participation with, organisational employee mental health support will be considered a facilitator. Any factor that limits or restricts the development, implementation, adoption, uptake of or participation with organisational employee mental health support will be considered as a barrier. The same factor may be both a barrier and a facilitator.

Information sources

The following electronic databases were searched between 18th – 23rd February 2022 (and will be re-run six weeks before review completion): AMED, CINAHL, Cochrane Central Register of Controlled Trials and the Cochrane Database of Systematic Reviews via the Cochrane Library,

For peer review only - http://bmjopen.bmj.com/site/about/guidelines.xhtml

EMBASE, EMCARE, HMIC, Medline, PsycINFO, Scopus and Web of Science . An example search strategy for Medline is presented in Appendix 1. Searches were tailored to each database using the Polyglot Search Translator[24] and conducted using keywords and relevant theasai such as MeSH and EMTREE. To ensure that all the available and relevant research is captured, grey literature will also be sought from the OpenGrey, MedNar and ProQuest databases and through the webpages of industry and charitable organisations active in supporting EMS ambulance employee mental health. A full list of webpages to be manually searched will be developed by the research team and will include sites such as the Global Ambulance Leadership Alliance (which covers the UK, US, Canada, and Australasia), The Ambulance Staff Charity (UK), the Royal Foundation, and the mental health charity, Mind. The reference lists of all studies selected for critical appraisal will be hand searched for further material for inclusion. The searches will be re-run six weeks prior to the final analyses to identify and retrieve any other studies for inclusion.

Supplemental material

See Appendix 1: Example Medline OvidSP search strategy

Study Records

Data management

References identified from electronic and hand searches, including title and abstracts, will be imported into Mendeley citation manager software and any duplicates removed.

Selection process

Two reviewers will independently screen a subset (10%) of titles and abstracts. Full-text screening will be based upon a PICoT concept:

- ***Population***: Adults (18+) employed by government/state commissioned EMS ambulance services.

For peer review only - http://bmjopen.bmj.com/site/about/guidelines.xhtml

- *phenomena of Interest*: Types of organisational interventions offered to support ambulance staff mental health and any barriers and/or facilitators to utilising such support.
- ***Co**ntext*: Government/state commissioned prehospital EMS ambulance organisations.
- ***T**ypes of studies study design*: All types of research studies.

Studies scoring 4/4 for all the above criteria will be included. Any reviewer uncertainty will be rated as 'unsure' and discussed by the independent reviewers with reference to the full text if required. If not resolved through reviewer discussion, disagreements will be settled through discussion with an independent third reviewer. The inter-rater reliability of consensus will be calculated.

Data extraction process

To identify papers for inclusion the full text of remaining studies will be retrieved and screened. Again, the inter-rater reliability will be calculated to ensure consistency and clarity. From this final selection, all potentially relevant data will be extracted and collated in an Excel spreadsheet including:

- Primary author
- Publication details
- Country of study
- Study methods
- Setting
- Sample characteristics (sample size, age range, EMS job role)
- Phenomenon of interest (self-reported barriers and/or facilitators)
- Intervention (where relevant)

Outcomes measured will include:

- Primary outcome measures (self-reported barriers and/or facilitators)

For peer review only - http://bmjopen.bmj.com/site/about/guidelines.xhtml

- Assessment tool names
- Reported statistics
- Reported significance levels
- Reported effect sizes
- Secondary outcome measures
- Relevant findings

To ensure sufficient detail capture to enable replication, any described intervention content will be extracted using Hoffman et al's[25] template for intervention description and replication (TIDieR) checklist. If data are missing or additional information is required, we will contact authors by email as per Cochrane recommendations and document the frequency of contact and authors' responses. [26]. Search results will be reported in full and presented in a Preferred Reporting Items for Systematic Reviews and Meta-analyses (PRISMA) flow diagram.

Quality assessment

The quality, alongside the trustworthiness, relevance, and findings of each of the studies identified for final selection will be assessed by two independent reviewers using two rating checklists (Standard Quality Assessment Checklists) developed by Kmet, Cook and Lee.[27] One checklist is designed to assess the quality for quantitative studies (and will also be applied to the quantitative components of mixed-methods studies) and the other for qualitative studies (which will also be applied to the qualitative components of mixed-methods studies). Each checklist item will be rated on a quality scale from 0–2:

- criteria not met = 0
- criteria partially met = 1
- criteria fully met = 2

Any included grey literature will be assessed using Tyndall's[28] 'Authority, Accuracy, Coverage, Objectivity, Date, Significance' (AACODS) checklist. Reviewer discrepancies will be resolved through discussion and when necessary, consultation with the third reviewer. All study types

For peer review only - http://bmjopen.bmj.com/site/about/guidelines.xhtml

will be included in this review, regardless of methodological quality, since it is anticipated that the availability of high-quality evidence will be limited. However, a sensitivity analysis will be conducted by testing whether removing any studies rated zero for methodological quality from the analysis changes the thematic results. Critical appraisal results will be displayed in a pre-determined assessment of methodological quality table. The narrative synthesis will include a summary of the relative impact of missing data and of methodological flaws on the findings.

Data synthesis

Mixed-methods systematic reviews are an emerging field of enquiry, useful for enhancing the credibility of findings. This is particularly important for this review as although quantitative evidence suggests that ambulance staff report high rates of mental ill health and want organisational support,[9,29] evidence from qualitative studies indicates that negative experiences and perceptions of such support can affect the acceptability of utilising support.[30] By using a mixed-methods approach, both the experience *and* effectiveness of organisational support initiatives can be captured; factors vital for informing the research question. The mixed-methods procedure will follow Joanna Briggs Institute guidance for a convergent integrated approach.[31] This involves transforming extracted data from quantitative papers (and quantitative aspects of mixed-methods papers) by qualitizing (creating a textual description) quantitative findings. This enables findings from all studies to then be combined during the analysis phase. It is anticipated that data from the included studies will be heterogeneous since they are likely to include different approaches to design and use of different outcome measures. Heterogeneity will be determined by summarising:

- Population characteristics (e.g., sample-size, age, type of mental health problem/disorder)
- Methodological approach (e.g., qualitative, survey, experiment)
- Assessment (the measures used to assess staff perceptions of organisational support, barriers or facilitators where relevant)
- Intervention characteristics (e.g., type of intervention, frequency, duration, uptake)

For peer review only - http://bmjopen.bmj.com/site/about/guidelines.xhtml

It is therefore unlikely that it will be possible to undertake a meta-analysis. Instead, a narrative review and synthesis approach will be taken by conducting inductive thematic analysis, using NVivo software and data from the excel data extraction sheets in the following steps:

1. Key data (data from the results sections of included papers) and quotations will be transposed from data extraction sheets to NVivo for coding by two reviewers, who will agree a coding structure for coding of participant data. The third reviewer will arbitrate any conflict.

2. Using the agreed upon coding structure, two reviewers will undertake thematic analysis of the coded data and will meet regularly to ensure the coding structure is appropriate and can be applied to the conclusions being drawn from the identified themes.

3. Factors impacting on participation with EMS organisational employee mental health support, with a focus on information from studies relating to employee experiences and/or perceptions of barriers against, or facilitators to, accessing and utilising support, will be synthesised in this systematic review (there will be no minimum number of studies).

4. A combined narrative (descriptive) synthesis will be used following Campbell et al's[20] SWiM guideline.

5. The certainty of evidence will also be synthesised using #27 quality checklist.

Amendments

If any protocol amendments are required, the date, description and rationale will be made available on the PROSPERO registration.

For peer review only

For peer review only - http://bmjopen.bmj.com/site/about/guidelines.xhtml

Patient and public involvement

To enhance the meaningfulness and robustness of findings, an EMS staff reference group and an EMS specific patient involvement group in the UK reviewed and supported the development and design of this protocol. These groups will review and provide an employee and public perspective on the interpretation of the findings and will support dissemination.

Ethics and dissemination

Ethical approval is not required because only available published data will be analysed and this is a protocol for a systematic review. Findings will be disseminated through publication in a relevant peer reviewed journal. The findings will also be communicated at research conferences, symposia, congresses and via social media to ensure dissemination to a wide range of interested parties.

Discussion

EMS employee mental wellbeing can influence the care given to patients. A number of initiatives are provided to support EMS employee mental health, although evidence suggests that some staff don't seek help or feel unable to disclose their mental health status when needed. With this in mind, a strength of this systematic review will be the presentation of barriers and facilitators specific to the uptake of employee mental health support in the EMS context identified through robust, replicable methods and critical appraisal of the available literature. Limitations will be addressed through transparent reporting and appraisal of study quality the involvement of EMS staff in the development of the inclusion and exclusion criteria, and by grading of the quality of the studies included.

For peer review only - http://bmjopen.bmj.com/site/about/guidelines.xhtml

Acknowledgements

We acknowledge the help and support of senior librarians Laura Coysh, Plymouth Discovery Library, Derriford Hospital, Plymouth and Karine Barker, Radcliffe Science Library, Bodleian libraries, University of Oxford. We are grateful for the funding provided by NHS England and NHS Horizons and for the support of South Western Ambulance NHS Foundation Trust.

Contributors

This study concept and design were conceived by authors SJ, JW and KS. SJ and JW drafted this manuscript with support from KS and LB who reviewed and edited the final version. All have approved the final submission.

Conflict of Interests statement

None declared

Funding statement

This work was supported by NHS England and NHS Horizons 'Project A' and by South Western Ambulance NHS Foundation Trust as part of SJ's DPhil in Experimental Psychology with the University of Oxford. JW is supported by MQ (CQRO1260), the Wellcome Trust (00070), and Oxford Health NIHR Biomedical Research Centre.

For peer review only

References

1 Roy D, Weyman A, Nolan P. Self-preservation comes at a cost: Why British National Health Service paramedics might be choosing a healthier, but poorer, retirement. *SAGE open Med* 2020;**8**. doi:10.1177/2050312120901545

2 Petrie K, Milligan-Saville J, Gayed A, *et al.* Prevalence of PTSD and common mental disorders amongst ambulance personnel: a systematic review and meta-analysis. *Soc Psychiatry Psychiatr Epidemiol* 2018;**53**:897–909. doi:10.1007/s00127-018-1539-5

3 Stevelink SAM, Pernet D, Dregan A, *et al.* The mental health of emergency services personnel in the UK Biobank: a comparison with the working population. *Eur J Psychotraumatol* 2020;**11**. doi:10.1080/20008198.2020.1799477

4 Mind. Behind the Mask: How the coronavirus pandemic impacted the mental health of emergency responders. London: 2021. https://www.mind.org.uk/media/7246/blue-light-behind-the-mask-report-en-2021.pdf (accessed 13 Jun 2022).

5 James S. Prevention of Suicide in the Ambulance Service What We Know Health Education England 2 What We Know. London: 2021. https://aace.org.uk/wp-content/uploads/2021/05/CCS1120525712-003_Prevention_of_Suicide_in_the_Ambulance_Service_What_we_know_Web-Accessible.pdf (accessed 4 Jan 2022).

6 Mars B, Hird K, Bell F, *et al.* Suicide among ambulance service staff: a review of coroner and employment records. *Br Paramed J* 2020;**4**:10–5. doi:10.29045/14784726.2020.12.4.4.10

7 Wagner SL, White N, Regehr C, *et al.* Ambulance Personnel: Systematic Review of Mental Health Symptoms. *Traumatology (Tallahass Fla)* Published Online First: 2020. doi:10.1037/trm0000251

8 Maunder RG, Halpern J, Schwartz B, *et al.* Symptoms and responses to critical incidents in paramedics who have experienced childhood abuse and neglect. *Emerg Med J* 2011;**29**:222–7. doi:10.1136/emj.2010.099838

9 Mind. *Mental Health in the Emergency Services: Our 2019 Survey Results - Ambulance*

For peer review only - http://bmjopen.bmj.com/site/about/guidelines.xhtml

Service. London: : Mind 2019.

10 Willis E, Lawn S, Roberts L, *et al.* The impact of emergency call taking on the mental health and wellbeing of ambulance call-takers: A systematic thematic narrative of qualitative research. *Australas J Paramed* 2020;**17**:1–11. doi:10.33151/ajp.17.801

11 South Western Ambulance NHS Foundation Trust. South Western Ambulance Service NHS Foundation Trust Annual Report and Accounts 1 April 2019 - 31 March 2020. Exeter: 2020. https://www.swast.nhs.uk/assets/1/swestamb_annual_report_with_accounts_2019-20.pdf (accessed 29 Apr 2022).

12 Gouweloos-Trines J, Tyler MP, Giummarra MJ, *et al.* Perceived support at work after critical incidents and its relation to psychological distress: a survey among prehospital providers. *Emerg Med J* 2017;**34**:816–22. doi:10.1136/emermed-2017-206584

13 Stevenson D, Farmer P. Thriving at work. London: 2017. https://assets.publishing.service.gov.uk/government/uploads/system/uploads/attachment_data/file/658145/thriving-at-work-stevenson-farmer-review.pdf

14 World Health Organization. Preventing Suicide: A global imperative. 2014. https://apps.who.int/iris/bitstream/handle/10665/131056/9789241564779_eng.pdf;jsessionid=B4E631D830D2F3E40760A7E2416C01BF?sequence=1 (accessed 10 Mar 2022).

15 Walumbwa FO, Muchiri MK, Misati E, *et al.* Inspired to perform: A multilevel investigation of antecedents and consequences of thriving at work. *J Organ Behav* 2018;**39**:249–61. doi:10.1002/job.2216

16 Hampson E, Jacob A. Mental health and employers; Refreshing the case for investment. London: 2020. https://www2.deloitte.com/content/dam/Deloitte/uk/Documents/consultancy/deloitte-uk-mental-health-and-employers.pdf

17 House of Commons. Workforce burnout and resilience in the NHS and social care Second Report of Session 2021-22 Report, together with formal minutes relating to the report. London: 2021. www.parliament.uk/hsccom (accessed 2 Jan 2022).

18 NHS Improvement. Operational productivity and performance in English NHS Ambulance

For peer review only - http://bmjopen.bmj.com/site/about/guidelines.xhtml

Trusts: Unwarranted variations. London: 2018. https://improvement.nhs.uk/documents/3271/Operational_productivity_and_performance_NHS_Ambulance_Trusts_final.pdf (accessed 7 Mar 2022)

19 Shamseer L, Moher D, Clarke M, *et al.* Preferred reporting items for systematic review and meta-analysis protocols (PRISMA-P) 2015: elaboration and explanation. *BMJ* 2015;**350**:g7647. doi:10.1136/bmj.g7647

20 Campbell M, McKenzie JE, Sowden A, *et al.* Synthesis without meta-analysis (SWiM) in systematic reviews: reporting guideline. *BMJ* 2020;**368**. doi:10.1136/BMJ.L6890

21 NHS Employers. Agenda for Change: NHS TERMS AND CONDITIONS OF SERVICE HANDBOOK. Leeds: 2004. https://www.nhsemployers.org/sites/default/files/2021-06/tcs-handbook-version-2004.pdf

22 National Highway Traffic Safety Administration. EMS Workforce for the 21st Century: A National Assessment. Washington, D.C.: 2008. https://www.google.com/url?sa=t&rct=j&q=&esrc=s&source=web&cd=&ved=2ahUKEwjFo8G6lbT2AhWPa8AKHQ6sDZ0QFnoECAsQAQ&url=https%3A%2F%2Fwww.ems.gov%2Fpdf%2Fresearch%2FStudies-and-Reports%2FNational_Workforce_Assessment.pdf&usg=AOvVaw3bI-r62sS-46Z8qX7F-c7C

23 ACT Parliamentary Counsel. Emergencies Act 2004: A2004-28. Canberra: 2004. https://www.legislation.act.gov.au/DownloadFile/a/2004-28/current/PDF/2004-28.PDF

24 Clark J, Carter M, Honeyman D, Cleo G, Auld Y, Booth D, Condron P, Dalais C, Dern S, Linthwaite B, May N, Munn J, Ramsay L, Rickett K, Rutter C, Sanders S, Sondergeld P, Wallin M BE. The Polyglot Search Translator (PST): evaluation of a tool for improving searching in systematic reviews: a randomised cross-over trial. In: *Abstracts of the 25th Cochrane Colloquium*. Edinburgh: : Cochrane Database of Systematic Reviews 2018. doi:10.1002/14651858.CD201801

25 Hoffmann TC, Glasziou PP, Boutron I, *et al.* Better reporting of interventions: template for intervention description and replication (TIDieR) checklist and guide. *BMJ* 2014;**348**. doi:10.1136/BMJ.G1687

26 Young T, Hopewell S. Methods for obtaining unpublished data. Cochrane.

For peer review only - http://bmjopen.bmj.com/site/about/guidelines.xhtml

2011.https://www.cochrane.org/MR000027/METHOD_methods-for-obtaining-unpublished-data (accessed 4 Aug 2022).

27 Kmet LM, Cook LS, Lee RC. Standard Quality Assessment Criteria for Evaluating Primary Research Papers from a Variety of Fields. Edmonton: 2004. doi:10.7939/R37M04F16

28 Tyndall J. AACODS Checklist. Adelaide: 2010. https://dspace.flinders.edu.au/xmlui/bitstream/handle/2328/3326/AACODS_Checklist.pdf?sequence=4&isAllowed=y (accessed 3 May 2022).

29 Dropkin J, Moline J, Power PM, *et al.* A qualitative study of health problems, risk factors, and prevention among Emergency Medical Service workers. *Work* 2015;**52**:935–51. doi:10.3233/wor-152139

30 Johnston S, Wild J, Sanderson K, *et al.* Perceptions and experiences of mental health support for ambulance employees. *https://doi.org/1012968/jpar2022147287* 2022;**14**:287–96. doi:10.12968/JPAR.2022.14.7.287

31 Lizarondo L, Stern C, Carrier J, *et al.* Chapter 8: Mixed Methods Systematic Reviews. In: *JBI Manual for Evidence Synthesis*. JBI 2020. doi:10.46658/JBIMES-20-09

For peer review only

Appendix 1: Medline search strategy

A draft search strategy in Medline for the following mixed-methods systematic review:

Why do ambulance employees (not) seek organisational help for mental health support?: A mixed-methods systematic review protocol of organisational support available and barriers/facilitators to uptake

Keywords

Emergency Medical Services; Mental Health; Organisational culture; Paramedical personnel; Systematic review

Search strategy

(Title/Abstract) (Medline):

1. “pre hospital"
2. pre-hospital
3. prehospital
4. paramedic*
5. ambulance*
6. aeromedical
7. “Aviation medicine”
8. HEMS
9. helicopter ADJ5 emergenc*
10. EMT
11. "emergency medical" ADJ technician*
12. ECA
13. "emergency care" ADJ assistant*
14. "Emergency call" ADJ (handler* OR operator*)
15. "Emergency dispatch*"

For peer review only - http://bmjopen.bmj.com/site/about/guidelines.xhtml

16. #1 OR #2 OR #3 OR #4 OR #5 OR #6 OR #7 OR #8 OR #9 OR #10 OR #11 OR #12 OR #13 OR #14 OR #15

---

17. anxiety
18. depress*
19. ptsd
20. "post-traumatic stress"
21. "post traumatic stress"
22. burnout
23. "burn out"
24. "self-harm"
25. self harm
26. "self injur*"
27. self-injur*
28. "self mutilat*"
29. self-mutilat*
30. distress
31. "mental health"
32. "mental illness*"
33. well-being
34. wellbeing
35. stress* suicid*
36. "critical incident stress"
37. #17 OR #18 OR #19 OR #20 OR #21 OR #22 OR #23 OR #34 OR #25 OR #26 OR #27 OR #28 OR #29 OR #30 OR #31 OR #32 OR #33 OR #34OR #35 OR #36

---

38. "occupational health"
39. "occupational mental health"
40. "occupational support"
41. "psychological support"
42. "psychological help"

43. help-seeking
44. help ADJ3 seeking
45. signposting
46. "employee assistance"
47. "employee support"
48. resilience
49. organi?ation* ADJ3 (support OR assistance)
50. work* ADJ3 (support OR assistance)
51. manager* ADJ3 (support OR assistance)
52. "crisis intervention"
53. downtime
54. surveil*
55. monitor*
56. #38 OR #39 OR #40 OR #41 OR #42 OR #43 OR #44 OR # 45 OR #46 OR #47 OR #48 OR #49 OR # 50 OR #51 OR #52 OR # 53 OR # 54 OR #55

57. #16 AND #37 AND #56

w only

PRISMA-P (Preferred Reporting Items for Systematic review and Meta-Analysis Protocols) 2015 checklist: recommended items to address in a systematic review protocol*

Section and topic	Item No	Checklist item	Section
**ADMINISTRATIVE INFORMATION**			
**Title: Why do ambulance employees (not) seek organisational help for mental health support? A mixed-methods systematic review protocol**			
Identification	1a	Identify the report as a protocol of a systematic review	Title p.1
Update	1b	If the protocol is for an update of a previous systematic review, identify as such	n/a
Registration	2	If registered, provide the name of the registry (such as PROSPERO) and registration number	Abstract p.3 & Methods p.7
Authors:			
Contact	3a	Provide name, institutional affiliation, e-mail address of all protocol authors; provide physical mailing address of corresponding author	Title page p.1
Contributions	3b	Describe contributions of protocol authors and identify the guarantor of the review	Contributor statement p.16
Amendments	4	If the protocol represents an amendment of a previously completed or published protocol, identify as such and list changes; otherwise, state plan for documenting important protocol amendments	n/a
Support:			
Sources	5a	Indicate sources of financial or other support for the review	Funding statement p.16
Sponsor	5b	Provide name for the review funder and/or sponsor	
Role of sponsor or funder	5c	Describe roles of funder(s), sponsor(s), and/or institution(s), if any, in developing the protocol	
**INTRODUCTION**			
Rationale	6	Describe the rationale for the review in the context of what is already known	Introduction pp.5-6
Objectives	7	Provide an explicit statement of the question(s) the review will address with reference to participants, interventions, comparators, and outcomes (PICO)	Aim and objectives pp.6-7 & Selection process p.10
**METHODS**			
Eligibility criteria	8	Specify the study characteristics (such as PICO, study design, setting, time frame) and report characteristics (such as years	Methods

For peer review only

		considered, language, publication status) to be used as criteria for eligibility for the review	pp.7-10
Information sources	9	Describe all intended information sources (such as electronic databases, contact with study authors, trial registers or other grey literature sources) with planned dates of coverage	Methods pp.9-10, & Data extraction p.12
Search strategy	10	Present draft of search strategy to be used for at least one electronic database, including planned limits, such that it could be repeated	Supplementary appendix 1
Study records:			
Data management	11a	Describe the mechanism(s) that will be used to manage records and data throughout the review	Data management pp.10-11
Selection process	11b	State the process that will be used for selecting studies (such as two independent reviewers) through each phase of the review (that is, screening, eligibility and inclusion in meta-analysis)	Data management/ synthesis pp.10-14
Data collection process	11c	Describe planned method of extracting data from reports (such as piloting forms, done independently, in duplicate), any processes for obtaining and confirming data from investigators	Data management/ synthesis pp.10-14
Data items	12	List and define all variables for which data will be sought (such as PICO items, funding sources), any pre-planned data assumptions and simplifications	Data management/ synthesis pp.10-14
Outcomes and prioritization	13	List and define all outcomes for which data will be sought, including prioritization of main and additional outcomes, with rationale	Data synthesis pp.10-14
Risk of bias in individual studies	14	Describe anticipated methods for assessing risk of bias of individual studies, including whether this will be done at the outcome or study level, or both; state how this information will be used in data synthesis	Quality assessment & data synthesis pp.12-14
Data synthesis	15a	Describe criteria under which study data will be quantitatively synthesised	
	15b	If data are appropriate for quantitative synthesis, describe planned summary measures, methods of handling data and methods of combining data from studies, including any planned exploration of consistency (such as I^2, Kendall's τ)	Data synthesis pp.12-14
	15c	Describe any proposed additional analyses (such as sensitivity or subgroup analyses, meta-regression)	
	15d	If quantitative synthesis is not appropriate, describe the type of summary planned	
Meta-bias(es)	16	Specify any planned assessment of meta-bias(es) (such as publication bias across studies, selective reporting within studies)	Quality assessment pp.12-13
Confidence in cumulative evidence	17	Describe how the strength of the body of evidence will be assessed (such as GRADE)	Quality assessment pp.12-13

*** It is strongly recommended that this checklist be read in conjunction with the PRISMA-P Explanation and Elaboration (cite when available) for important clarification on the items. Amendments to a review protocol should be tracked and dated.** **The copyright for PRISMA-P (including checklist) is held by the PRISMA-P Group and is distributed under a Creative Commons Attribution Licence 4.0.**

For peer review only

From: Shamseer L, Moher D, Clarke M, Ghersi D, Liberati A, Petticrew M, Shekelle P, Stewart L, PRISMA-P Group. Preferred reporting items for systematic review and meta-analysis protocols (PRISMA-P) 2015: elaboration and explanation. BMJ. 2015 Jan 2;349(jan02 1):g7647.

For peer review only
